# The recovery and retraction of memories of abuse: a scoping review

**DOI:** 10.3389/fpsyg.2025.1498258

**Published:** 2025-02-05

**Authors:** Henry Otgaar, Ivan Mangiulli, Chunlin Li, Marko Jelicic, Peter Muris

**Affiliations:** ^1^Faculty of Psychology and Neuroscience, Maastricht University, Maastricht, Netherlands; ^2^Faculty of Law and Criminology, Catholic University of Leuven, Leuven, Belgium; ^3^Dipartimento Di Scienze Della Formazione, Psicologia, Comunicazione, University of Bari “Aldo Moro”, Bari, Italy

**Keywords:** retractor, memory, trauma, memory wars, false memory, recovered memory

## Abstract

People who claim to have been abused sometimes retract these claims at a later point in time. Research on these so-called ‘retractors’ might provide critical insights into the processes involved in the recovery and retraction of traumatic memories. However, the literature on this topic is highly diverse in terms of, for example, methodology. Hence, the aim of the current scoping review was to amass the available literature on retractors and identify key themes. We identified 17 articles on the topic of retractors ranging from empirical studies to critical commentaries. A central theme that arose from the literature was the influence of therapy in the recovery of potentially false memories. That is, retractors noted that therapists frequently believed that they harboured unconscious repressed memories of abuse which had to be recovered during therapy. Furthermore, retractors repudiated their claims of abuse for various reasons such as physical evidence implying that their memory was false. Also, retraction took longer that the initial recovery of memories of abuse. Finally, after recantation, retractors’ memories varied considerably in terms of belief and recollection of the traumatic event with some accounts qualifying as nonbelieved memories. This review offers critical knowledge of a rather understudied population providing further insight in how traumatic events can sometimes be misremembered.

## Introduction

On the 5^th^ of September 1991, Melvin Quinney was convicted to 20 years imprisonment^1^. His conviction was heavily based on the testimony of his 10-year-old son, John Parker. John testified that his father had sexually abused him. However, his testimony was not based on his own memories but the result of family, friends, and psychologists of John suggesting to him that his father had abused him. Although John initially denied being abused by his father, these suggestions eventually made him (falsely) claim that he was abused. Many years later, in 2014, John retracted this memory after realizing that his memories were false, and that his father had not abused him. From 2014 onwards, John aimed to exonerate his father and succeeded in 2023.

John is known as a retractor, a term to describe individuals who once reported to have been abused but later repudiated their claim stating that they were not abused (e.g., De Rivera, 2000). The issue of retractors has received attention in discussions on the authenticity of recovered memories in therapy (e.g., Felstead and French, 2022; Ost et al., 2001). Specifically, some retractors’ cases pertain to individuals who suffered from mental health complaints and sought therapeutic help. In these cases, therapists sometimes suggested to them that their mental health problems were the result of repressed memories of abuse. As a result of these suggestions, some clients started to recover memories of abuse that they did not have before treatment (e.g., Loftus and Ketcham, 1994; Otgaar et al., 2019).

A key question surrounding these recovered memories is whether they refer to genuine memories. Interestingly, retractors play a unique role in discussions surrounding the authenticity of recovered memories as they initially claimed to have been abused and later retracted these claims. Hence, some scholars have suggested that these retracted claims should be viewed with a cautionary eye as they do not say anything about whether the abuse happened or not (Reviere, 1997; Singer, 1997; Vos, 2024). Statements of retractors might provide critical insights in the authenticity of recovered memories and more broadly, the impact of trauma on memory. In the current review, we have assembled the literature on retractors. We will first start with an overview on the topic of repressed and recovered memories and will then focus on retractors.

## Repressed and recovered memories

### Repressed memory

The topic of recovered memories has garnered much attention in debates regarding the existence of the controversial concept of repressed memory. Repressed memory refers to a traumatic memory that, because of its painful and overwhelming nature, is unconsciously stored in pristine form and is inaccessible (Freud, 1962; Loftus and Ketcham, 1994). In the 1990s, a heated debate took place on the question whether traumatic memories could be unconsciously repressed. Much of this debate originated from legal cases in which patients recovered memories of abuse in therapy that they were unaware of before therapy and then sometimes falsely accused family members (e.g., Manzanero and Morales-Valiente, 2024; Patihis et al., 2014). According to some therapists, the patients had repressed the trauma for many years and psychological treatment helped them to uncover the repressed memory. However, memory researchers warned that these therapy-induced recovered memories might in fact be false memories (i.e., memories for events/details that were not experienced; Loftus, 2005) evoked by the suggestive nature of the therapy. This debate is also known as the memory wars (e.g., Loftus, 1993).

Researchers have expressed criticisms concerning the existence of repressed memory (e.g., Dodier et al., 2024; McNally, 2024; Otgaar et al., 2019). Specifically, the idea of repressed memory stands in contrast with a bulk of research showing that traumatic and stressful events are generally well-remembered (e.g., Shields et al., 2017), even after a long delay (e.g., Goldfarb et al., 2019). Also, the concept of repressed memory is sometimes confused with normal memory mechanisms such as ordinary forgetting or ways to cope with trauma such as not wanting to think or talk about the trauma (e.g., McNally, 2005). Another critique of repressed memory is that it is an unfalsifiable construct, meaning that it cannot be empirically tested. Specifically, because repressed memory is thought to be unconsciously stored and is inaccessible, it is not possible to access it and subject it to scientific investigation (Otgaar et al., 2022a; see De Brigard, 2024; Patihis, 2023 for other critiques).

Importantly, although some scholars have declared the memory wars to be over (Barden, 2016; McHugh, 2003; Paris, 2012), empirical evidence shows that the controversial phenomenon of repressed memory continues to thrive in legal, clinical, and academic spheres (Battista et al., 2023; McNally, 2024; Otgaar et al., 2019). The main concern is that therapists believing in repressed memory might suggest to their patients that their symptoms are the consequence of hidden or repressed memory of trauma, which might then lead to the creation of false recovered memories (Otgaar et al., 2019). Recent studies have indeed confirmed that therapists sometimes discuss the existence of repressed memory with their patients. For example, Zappalà et al. (2024) interviewed Italian cognitive behavioural therapists and trainees (*N* = 402) about their therapeutic practices and in specific their belief in repressed memory. Eighty-three percent (*n* = 334) endorsed the belief that traumatic memories are often inaccessible due to their painful nature. In addition, half of the therapists (*n* = 98) indicated that they sometimes discussed the existence of unconscious traumatic memories with their patients and about 60% (*n* = 126) even reported that they encountered patients recovering memories that they were unaware of before therapy. Similar results were obtained in a German sample of psychotherapists (Schemmel et al., 2024). These results suggest that the concept of repressed memory is still widely accepted among clinicians, but even worse that suggestive therapeutic practices continue to be used, potentially fomenting the formation of false recovered memories.

### Recovered memories

A major aspect of the memory wars pertains to the generation of recovered memories in therapy. Recovered memories are memories that people are unaware of until they retrieve them (Dodier et al., 2023). The worry here is that these recovered memories might be instilled due to suggestive therapy, which would imply that these recovered memories are in fact false memories (see also Lynn et al., 2015). However, recovered memories are not by definition false memories. They can refer to authentic experiences as well (McNally and Geraerts, 2009). For example, people who have been traumatized sometimes talk about their experiences but many years later do not remember these conversations anymore. When they recover these traumatic experiences, they have a feeling that they were completely unaware of these memories, but in fact did discuss these memories indicating that they had been aware of these memories many years ago (McNally and Geraerts, 2009).

Recovered memories can also occur to people reinterpreting earlier experiences (McNally and Geraerts, 2009; Patihis et al., 2019). Specifically, during sexual abuse, a child might not understand that he or she is being victimized and hence, may not have any strong negative emotions towards the event. However, many years later, this specific person realizes that the event concerned abuse and reinterprets the event as emotionally negative and traumatic. In such instances, people who recover such memories might subjectively report that they were unaware of the memory but in fact had these memories before but at a later point reinterpreted and reappraised them (Clancy, 2011).

For legal cases, truth finding is essential and thus it is imperative to know whether recovered memories refer to false or true memories. One way to investigate this issue is to examine the occurrence of recovered memories in different contexts (e.g., in or outside therapy). Patihis and Pendergrast (2019), for example, surveyed 2,326 US citizens from the general population and asked whether they ever underwent therapy. If this appeared to be the case, participants were asked whether the therapists discussed the existence of repressed memory and whether they recovered memories in therapy that they did not have prior to therapy. Nine percent reported that the therapists discussed the likelihood of repressed memories of abuse and 5% reported to have recovered memories of abuse of which they were unaware of before therapy (see also Dodier et al., 2019 for similar results in France).

These studies reveal the occurrence of recovered memories in therapy. However, recovered memories do not exclusively happen in therapy and can occur outside therapy as well. For example, Dodier and Patihis (2021) asked French participants (*N* = 3,346) whether they ever recovered a memory and in which context this happened (i.e., in or outside therapy). Two interesting findings emerged. First, about one-third of the participants who reported to have recovered memories indicated that they always had these memories but reinterpreted them as abusive at a later stage in life. Second, 90% of the recovered memories that the participants were previously unaware of were retrieved outside of therapy (due to discussions with peers and/or exposure to media related to abuse).

Other populations can also be tested to obtain a picture of the occurrence of recovered memories. As mentioned above, recent empirical work has demonstrated that 60% (*n* = 126) of surveyed therapists reported to have witnessed the phenomenon of recovered memories in their clinical practices (Schemmel et al., 2024). Another strategy that might reveal insights on the instigation of recovered memories during therapy is interviewing people who claim to have been falsely accused of abuse. McHugh et al. (2004) sent questionnaires to families in the US who were allegedly falsely accused of abuse. Families who participated (*N* = 1,847) indicated that 86% of the accusers were in therapy when the accusation was made. Furthermore, 92% of the accusations involved repressed memories. Recent German data showed that around three-quarters of the accusers were in therapy prior to or at the time of the accusation (Houben et al., 2024). Collectively, these data imply that potentially false recovered memories continue to occur in therapeutic settings. However, an often-overlooked group concerning this issue are the so-called ‘retractors’, a group that can provide further critical insights in the development of recovered memories and the repudiations of memories for trauma.

## Retractors

Retractors are individuals who once claimed to have been abused but at a certain point repudiate this claim (Lief and Fetkewicz, 1997). Perhaps one of the first public retractors was an adult woman, Lynn Price Gondolf (see Davis, 2005). Gondolf was treated for an eating disorder by a therapist who asked her about the possible existence of childhood sexual experience. She told the therapist about her uncle who had repeatedly raped her during her childhood years. However, the therapist found her symptoms so severe that he started to suggest that her parents had also abused her. Due to the repeated suggestions, Gondolf started to believe her parents had indeed abused her. The retraction happened many years later when Gondolf consulted another therapist and was involved in a drug-rehab program. By focusing on the present (rather than the past), she started to realize that her parents had not abused her. Because of shame, she did not have contact with her parents for two more years, but eventually they reconciled.

The literature regarding retractors is rather scattered. For example, different methodologies have been used (e.g., case studies, quantitative research; Davis, 2005; DeGloma, 2007) and articles have been published from different perspectives (e.g., psychological, sociological; see DeGloma, 2007; Ost et al., 2001). The observation that retractors once claimed to have been abused but later withdrew these claims begs several important questions. Specifically, one might wonder about the origin of the abuse-related memories before retraction. Some scholars have noted that many of these retractors have recovered their memories in therapy thereby questioning the authenticity of these memories (e.g., Nelson and Simpson, 1994). Apart from questioning the validity of the recovered memory, concerns have been raised on the authenticity of the retraction itself, arguing that a retraction does not necessarily mean that the recovered memories were false (e.g., Vos, 2024).

Furthermore, and perhaps more fundamentally, recanting reports of abuse raises questions about the effect of such retractions on the quality of the memory report. That is, the phenomenon of retraction of memories is reminiscent of research in the field of nonbelieved memories (Ost, 2017). Nonbelieved memories are memories of events for which the belief in the occurrence in those events is substantially reduced (e.g., Mazzoni et al., 2010; Otgaar et al., 2014). Most of our experiences are accompanied with a strong recollection and a strong belief in them. Nonbelieved memories are the exception showing the flexibility of how experiences are remembered. Interestingly, nonbelieved memories can be elicited due to external social feedback such as someone stating that a memory is incorrect (Scoboria et al., 2015). This implies that retracted memories can be instances of nonbelieved memories as well (Li et al., 2023; Ost, 2017).

## The current review

Taken together, research on retractors can contribute to the understanding of how traumatic experiences are recovered and remembered or forgotten. To gain insight into these processes, Lynn et al. (2023) recommended “[c]onducting systematic studies of “retractors” versus individuals who maintain a sense of a divided self that is based on recovered memories” (p.736). In line with their recommendation, we conducted a scoping review of the literature on retractors and explored key themes surrounding the memories of retractors.

## Method

### Literature search

**Figure 1:**
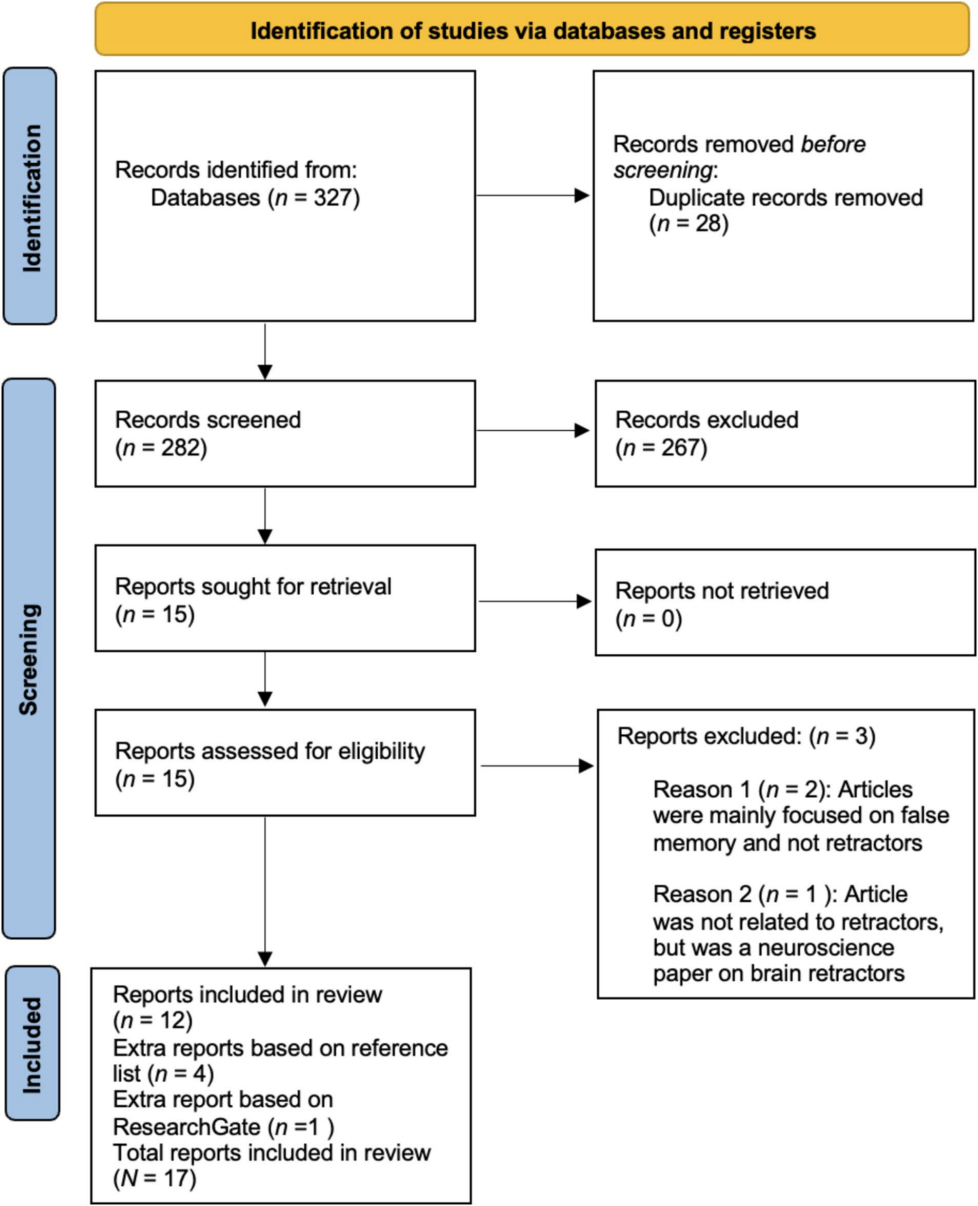
Identification of studies concerning retractors of abuse using PRISMA flowchart.

We used the Preferred Reporting Items for Systematic Reviews and Meta-Analyses (PRISMA; Page et al., 2021) workflow for our literature search (see Figure 1) and used the PRISMA-Scoping Review Checklist to guide our review (see https://osf.io/yt2ds). The following search terms were used: “Retractor” AND “Abuse” (everywhere), “Retractor” AND “trauma” (everywhere), “Retractor” AND “false memory” (everywhere), “Retractor” AND “repressed memory” (everywhere), “Retractor” AND “dissociative amnesia^2^” (everywhere). These search terms were applied to three databases (PsycInfo, Web of Science, and PubMed). Our search took place between March 5 and June 6, 2024. It led to the discovery of 327 papers. After removing duplicates, we had 282 papers that were screened.

Our inclusion criteria were (1) that the main topic of the papers should be on retractors of any abuse, (2) that the articles could be empirical studies, review, or discussion/commentary papers, (3) that the articles should be peer-reviewed, (4) that the articles should be published in English, and (5) that the articles should be published between 1990 and 2024. Exclusion criteria were that the papers had a main focus on false memory, repressed memory, trauma and memory and only briefly linked this work to retractors. The first and second authors independently screened the papers which led to the retrieval of 15 papers (inter-rater reliability: Cohen’s Kappa = 0.839, 95%CI [0.694, 0.994]; see https://osf.io/34zvq)^3^. Three of the retrieved papers were still excluded because they predominantly focused on false memory or were not specifically concerned with retractors of abuse. When inspecting the reference lists, four extra papers were found and in addition one recent paper (Vos, 2024) was discovered on ResearchGate. Altogether, our final sample consisted of 17 articles (see Table 1; see also https://osf.io/bqpuh).

**Table 1:** Included articles in the scoping review.

Authors	Title	Year	Journal	Type of article
Lief and Fetkewicz	Retractors of false memories: The evolution of pseudo-memories	1995	The Journal of Psychiatry and Law	Study
Nelson and Simpson	First glimpse: An initial examination of subjects who have rejected their recovered visualisations as false memories	1994	Issues in Child Abuse Accussations	Study
De Rivera,	The construction of false memory syndrome: The experience of retractors	1997	Psychological Inquiry	Study
Qin, Tyda, and Goodman	Retractors’ experiences: What we can and cannot conclude	1997	Psychological Inquiry	Commentary
Gudjonsson	False memory syndrome and the retractors: Methodological and theoretical issues	1997	Psychological Inquiry	Commentary
Reviere	Reflections on false memories, psychotherapy, and the question of “truth”	1997	Psychologicical Inquiry	Commentary
Singer	How recovered memory debates reduce the richness of human identity	1997	Psychologicical Inquiry	Commentary
De Rivera	Understanding persons who repudiate memories recovered in therapy	2000	Professional Psychology: Research and Practice	Study
Ost, Costall, and Bull	False confessions and false memories: a model for understanding retractors’ experiences	2001	The Journal of Forensic Psychiatry	Study
Ost, Costall, and Bull	A perfect symmetry? A study of retractors’ experiences of making and then repudiating claims of early sexual abuse	2002	Psychology, Crime and Law	Study
McHugh, Lief, Freyd, and Fetkewicz	From refusal to reconciliation—Family relationships after an accusation based on recovered memories	2004	The Journal of Nervous and Mental Disease	Study
Davis	Victim narratives and victim selves: False memory syndrome and the power of accounts	2005	Social Problems	Study
DeGloma	The social logic of false memories: Symbolic awakenings and symbolic worlds in survivor and retractor narratives	2007	Symbolic Interaction	Study
Ost	Adults’ retractions of childhood sexual abuse allegations: high-stakes and the (in)validation of recollection	2017	Memory	Study
Felstead and French	Dr James Ost’s contributions to the work of the British false memory society	2022	Memory	Review
Li, Otgaar, van Daele, Muris, Houben, and Bull	Investigating the memory reports of retractors regarding abuse	2023	The European Journal of Psychology Applied to the Legal Context	Study
Vos	What is the problem with retractors?	2024	European Journal of Trauma and Dissociation	Review

### Data extraction

We created a data file in which, for each paper, we included the following data points: (1) the authors, (2) title of the article, (3) year of publication, (4) journal, (5) type of publication, and (6) main result/conclusion (see also https://osf.io/wbqze). We created different data sheets for the inclusion of the papers. In the first sheet, we included the data of the 15 retrieved articles. In a second sheet, we inserted the data points of the four extra papers detected based on the reference lists. In a third sheet, we added the article that we found on ResearchGate. The first and second authors also used this data file for their screening.

## Results

### Type of articles

When examining which type of articles were published on the topic of retractors, it can be noted that 10 (58.8%) articles concerned empirical studies (either quantitative or qualitative), two (11.8%) articles concerned reviews, four (23.5%) papers were commentaries, and one (5.9%) article described a case study. Regarding the reviews, although one article was listed as a review in the journal, when reading the article, it could be better described as a perspective paper (Vos, 2024).

### Year of publication

**Figure 2:**
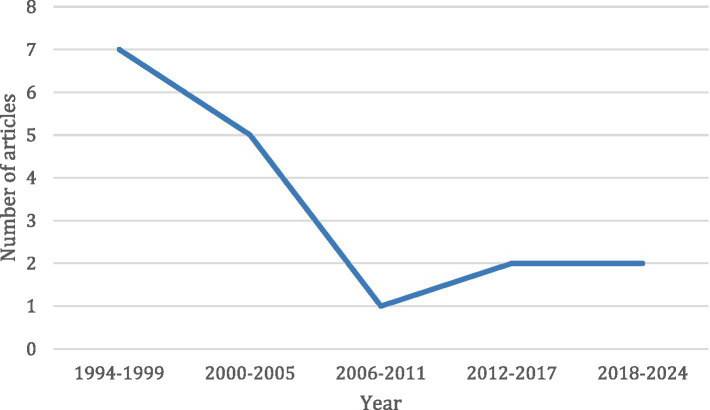
Number of publications per quinquennial on the topic of retractors. *Note*. The last period (2018–2024) is not 5 but 6 years.

The first identified article of our scoping review was published in 1994. Most articles regarding the topic were published in the period 1994–1999 (see Figure 2). After this period, there was a drop in publication until 2012; from that year on there was a slight increase of new articles being published on the topic of retractors.

### General content of articles

The included articles tackled different issues related to the topic of retractors. For example, several articles surveyed retractors and examined which factors led to the formation and retraction of memories concerning abuse (Li et al., 2023; Lief and Fetkewicz, 1997; Nelson and Simpson, 1994). One study examined families who were accused of abuse and examined whether some accusers could be classified as retractors (McHugh et al., 2004). Other studies looked at accounts of retractors and investigated the content of these accounts. To provide some examples, Ost et al. (2001) investigated the accounts of retractors and compared them with different types of false confessions. Davis (2005) assessed the use of symbolic language in retractors’ experiences such as stating that sexual abuse referred to game and play behavior. Furthermore, De Rivera (2000) examined the reasons why retractors originally started to believe and remember abuse.

There were also articles that provided a critical look towards the retraction of memories. In these articles, scholars argued that retractions of memories do not necessarily imply that the original memories were false (Reviere, 1997; Vos, 2024). Also, some scholars criticized earlier work on retractors (De Rivera, 1997) stating that this work provides a simplistic view on what can happen during therapy (Singer, 1997) or that this work cannot generalize to all cases of retractors (Qin et al., 1997).

### Detailed content of articles

When analyzing the different articles, it was evident that the articles could be grouped in literature related to (1) the formation of memories before retraction, (2) characteristics of retractors’ experiences, and (3) the aftermath of retraction. We will now discuss these issues in more depth.

#### The formation of memories before retraction

Several articles focused on how retractors created memories of abuse in the first place. A recurring theme in the reviewed studies is that therapy played a central role in the recovery of memories of abuse. Specifically, in one of the earliest studies, Nelson and Simpson (1994) sent questionnaires to and held telephone surveys in 20 people who identified themselves as retractors. It was found was that the large majority of retractors (*n* = 19; 95%) stated that their memories were recovered during the course of therapy, while one subject reported that the memory of abuse had evolved after reading a book entitled *“The Courage to Heal.”* (Bass and Davis, 1988) This book includes information concerning symptoms that are indicative of repressed memory of abuse. Importantly, all retractors stated that they did not have any memories before therapy or reading the book.

The central role of therapy is also evident in other studies. For example, Lief and Fetkewicz (1997) surveyed 40 retractors on their experiences. The vast majority of them reported that their memories were recovered during therapy (92.5%, *n* = 37). It is noteworthy that 33 (82.5%) retractors stated that their therapists suggested to them that they were abused before any memory was recovered. The respondents reported to have received a wealth of different therapeutic techniques such as age regression, guided imagery, dream interpretation, and hypnosis, with a noticeable 70% (*n* = 28) of the sample indicating that they had also read the aforementioned book “*The Courage to Heal.”* (Bass and Davis, 1988; see also Lynn et al., 2015).

Davis (2005) looked at retractors’ accounts published in, for example, newsletters, magazines, and websites and analyzed 81 accounts. This scholar observed that these accounts roughly followed a similar chronological order: A first phase describing how retractors entered therapy, a middle phase involving details on what happened during therapy, and a final phase how they left therapy and oftentimes reunited with family members. As can be seen in Davis’ analysis, therapy played a central role in the experiences of retractors.

Some studies aimed to find other common themes for why retractors started to form memories of abuse in therapy. De Rivera (1997) reasoned that therapy-induced recovered memories might arise due to different processes, which are described in two models called the mind-control model and the narrative model. According to the mind-control model, people might form false memories in therapy because they regard the therapist as an authority who they completely trust. Because of this trust, people will not make their own decisions and rely solely on what a therapist is suggesting to them. The narrative model contends that people constantly construct a story or narrative of their own identity. Thus, people with mental health problems might try to find the causes of their problems (e.g., the existence of repressed memory) and with the help of a therapist create a (false) narrative that abuse occurred in childhood (see also Lynn et al., 2015). De Rivera interviewed four retractors and argued that two of them had experiences that were more in line with the mind-control model, while the other two were reminiscent of the narrative model. Importantly, he argued that therapy was an important factor in all retractors’ experiences.

De Rivera (2000) followed up on this work with a larger sample of retractors (*N* = 56) and added one extra explanatory model to the investigation. That is, he reasoned that some experiences could be explained by role-enactment in which people adopted a certain role (e.g., survivor of abuse) and interpreted psychological symptoms in the light of this role. De Rivera detected that 23 retractors’ experiences were more in line with the mind-control model, 10 were in keeping with the narrative model, two were mainly reflecting role-enactment, whereas experiences of other retractors involved a combination of various models.

#### Characteristics of retractors’ experiences

We also identified studies that examined the characteristics of retractors’ experiences and linked them with other constructs. McHugh et al. (2004) surveyed families (*N* = 1,847) who were accused of abuse and found that a minority of the accusers retracted their claims (8%) with some of them first returning to their families before retraction. Furthermore, DeGloma (2007) compared accounts of self-identified victims of abuse with retractors’ accounts. He found that both accounts followed a similar pattern of storytelling. Specifically, according to DeGloma, both victims and retractors used symbolic terms in their accounts when referring to abuse. A recurrent symbolic topic in victims’ accounts is that perpetrators would have stated that the sexual interaction would belong to game and play behavior. Furthermore, in these accounts, victims frequently questioned whether what happened to them was perhaps a dream or fantasy.

Similar to victims of abuse, retractors also used symbolic terms in their accounts. One common thread was that they described therapists as indoctrinating them into believing that they were victimized using terms such as “mind-control,” “brain-washing,” or “mental torture.” Also, DeGloma found that retractors claimed that therapists started to behave as a new family, which later turned out to be the cause of their false memories. Furthermore, this scholar noted that therapists acted as authority figures that affected the memory of both victims and retractors.

Ost et al. (2001) argued that retractors’ experiences are similar to false confessions. More precisely, the argument was made that the context in which retractors created recovered memories bears similarities with the context in which false confessions occur. Three types of false confessions exist: voluntary (i.e., false confessions that arise voluntarily without any external suggestive and social pressure), coerced-compliant (i.e., false confessions arising due to external social pressure), and coerced-internalized (i.e., false confessions due to external social pressure and for which it is also believed that a crime is committed; Kassin and Wrightsman, 1985). Ost and colleagues found that retractors’ accounts can be categorized along the lines of these three different types of false confessions suggesting that the field of false confessions could act as a model to understand the phenomenon of retracted memories of abuse as well (see also Felstead and French, 2022).

Finally, Ost et al. (2002) surveyed 20 retractors and investigated the process of recovery and retraction of memories of abuse. Two key findings emerged from their study. First, retractors reported that they experienced more social pressure when recovering memories of abuse than when they retracted these memories. This is in contrast with the argument of some critics stating that social pressure was more present when retracting than recovering memories (e.g., Reviere, 1997). Second, it was found that the process of retraction took longer than the recovery of memories of abuse (see also Li et al., 2023).

#### The retraction

Several papers concentrated on processes that occur surrounding the retraction of memories of abuse. For example, Ost (2017) examined accounts of retractors and assessed which strategies retractors used to verify their memories (e.g., Wade et al., 2014) and what happened with retractors’ belief and recollection of the recovered memory. It was found that retractors used an amalgam of different memory verification strategies such as being presented with physical evidence (e.g., interpreting body memories (e.g., choking) as an indication of abuse) or being told by someone else that their experience was false. Ost also found that accounts of retractors varied in terms of their belief and recollection of the experience ranging from accounts being coded as nonbelieved memories and other accounts being categorized as a false belief. Interestingly, Li et al. (2023) also asked retractors to rate their belief and recollection before and after they repudiated their claims. For example, on average, retractors rated the strength of the belief in the occurrence of the event lower after (*M* = 2.61; *SD* = 2.06) than before retraction (*M* = 6.09, *SD* = 4.42; see also https://osf.io/t2fn9) while no statistical difference emerged for the strength of the recollection.

Ost (2017) and Li et al. (2023) also examined the reasons why belief in the experience was reduced. Specifically, they looked at different reasons for belief reduction such as social feedback, event plausibility and external evidence. Both studies found that external evidence such as media reports that casted doubt on the authenticity of the memory was the most important reason that retractors mentioned. Another interesting result was noted by Li et al. (2023) who examined the outcomes after retraction. They found that retractors reported that the repudiation also had some benefits. More precisely, the retractors stated that the retraction gave them a chance to rebuild their lives, making new relationships with family and friends, and no longer falsely accusing a person.

Furthermore, in De Rivera’s (1997) article, the accounts of four retractors are discussed and these also described the processes surrounding the retraction. In all accounts, the retraction did not happen immediately, meaning that the retractors sometimes had several occasions to reconsider the alleged abuse before realizing that it never occurred. For example, one retractor (De Rivera, 1997; RP1) started to express doubts that her father had abused her and eventually contacted her family for reconciliation. Although she reunited with her father, her sisters were still angry because of her accusations. For another retractor (De Rivera, 1997; RP2), the retraction started when she had a new therapist who questioned her narrative of being abused by her father. The retractor, however, challenged the therapist and was confident that her father had abused her. Following this, a pastor also confronted her stating that nothing had happened and eventually, she realized that she was not abused. After sending her father a card, they met and reunited.

A third retractor read about a legal case concerning problems in therapy that shared similarities with her own treatment (De Rivera, 1997; RP3). The reading of the case was a first indication that her narrative of her uncle abusing her might be wrong. When confronting her therapist, the therapist stated that her treatment differed extensively with what happened in the particular case. Because of problems in her marriage, she consulted a family therapist and eventually stopped seeing the first therapist. This eventually made her stop believing in the abuse and she partly reconciled with her family. The final retractor originally believed her father had abused her, but her father already passed away (De Rivera, 1997; RP4). With help of a friend, she started to understand that nothing actually happened. This case highlights the emotional complexity of retraction, where external support played a critical role in her re-evaluation of past events. Collectively, these examples reveal the complex process of retraction.

## Discussion

People who, in a certain period of their lives, claim to have developed recovered memories of abuse sometimes retract these claims at a later stage. This phenomenon raises several questions about the underlying reasons for the retraction, the fate of the original memories, and the nature of the retracted experiences. In this scoping review, we assembled all the literature available on such a topic and provided a critical overview of several key issues related to retractors of abuse. We will now discuss these issues and highlight what they tell us about the recovery and retraction of traumatic memories.

### Therapy and memory

A recurring element in the experiences of retractors was the role of therapy in the recovery of traumatic memories. An especially noticeable finding was that many retractors noted that their therapists suggested to them that they were abused and had repressed these memories. A wealth of research shows that such suggestions can lead to the creation of false (recovered) memories (Arce et al., 2023). Furthermore, our review revealed that such therapy-induced recovered memories could be explained by different kind of theoretical models such as the mind-control and narrative model. This finding is intriguing because it implies that therapists did not only play a suggestive role in exhuming repressed memories. It also shows that, for some retractors, being in therapy helped them to create a (false) narrative to explain why they suffered from mental health complaints.

The fact that therapy seems to be play a central role in the recovery of potentially false memories mirrors findings from related research. For example, Patihis and Pendergrast (2019) surveyed 2,326 US citizens from the general public and 9% of them indicated that the therapists suggested the likelihood of repressed memories of abuse and 5% reported to have recovered memories of abuse of which they were previously unaware of (see also Dodier et al., 2019). Furthermore, this finding is related to recent research showing that therapists themselves admitted to sometimes use suggestive practices during therapy and encounter recovered memories in their treatment setting (Schemmel et al., 2024; Zappalà et al., 2024).

Of course, these findings do not indicate that therapy is inherently suggestive, but they do show that suggestions during therapy might engender potentially false recovered memories. The finding that therapy was reported as a main cause for retractors’ recovered memories is also relevant of recent research into therapies that change autobiographical memory and might increase the risk for false memory formation (see also Phelps and Hofmann, 2019). One concrete example here is Eye Movement Desensitization and Reprocessing (EMDR) therapy (Shapiro, 1989). During this therapeutic intervention, patients are asked to retrieve a distressing memory, and at the same time need to perform a concurrent task. For example, patients need to follow the index finger of the therapist moving from left to right while retrieving the upsetting memory (Van den Hout and Engelhard, 2012). This procedure has been shown to reduce the vividness and emotionality of the distressing memory (Lee and Cuijpers, 2013; Houben et al., 2020a).

Although performing eye movements during therapy is effective in making traumatic memories less distressing, there is some research showing that this effect comes with a cost in the sense that it can fuel the formation of false memories (e.g., Houben et al., 2018; Houben et al., 2020b; Otgaar et al., 2021; but see also Van Schie and Leer, 2019). This finding combined with research showing strong beliefs in the existence of (Dutch) EMDR practitioners (Houben et al., 2021) suggests that recovered memories can occur in settings where EMDR is applied. Support for this assertion comes from recent research showing that EMDR has been associated with the occurrence of recovered memories (e.g., Patihis and Pendergrast, 2019). Taken together, our finding that therapy is a key factor in the development of recovered memories underscores the need for further research on the costs and benefits of therapeutic interventions on memory (see also Talwar et al., in press).

### Memory verification and retraction

In our review, we also encountered articles that focused on strategies that retractors used to verify the validity of their memories (Li et al., 2023; Ost, 2017). Such strategies are not exclusively exerted by retractors. On the contrary, in daily life, people also tend to use verification strategies to examine whether certain experiences really happened or not (Wade et al., 2014). Interestingly, while there is a considerable overlap in strategies that retractors use when compared with those used by students (e.g., Wade et al., 2014), a noticeable difference was that, for example, in retractors’ accounts they were *presented* with physical evidence casting doubt on the experiences while student participants noted to actively *search* for physical evidence to validate their memories (Ost, 2017; Wade et al., 2014). This might suggest that other people (e.g., family members) already doubted retractors’ claim thereby presenting retractors with evidence indicating that their claims were false. Alternatively, it might imply that retractors were highly convinced in the authenticity of the recovered memories and hence, did not actively seek for external evidence as much as, for example, student participants.

### Belief and recollection of retracted experiences

An interesting and rather recent line of research has been done on the consequences of retraction on the belief and recollection of the recovered memory (Li et al., 2023; Ost, 2017). This interest originated from research on nonbelieved memories showing that belief in the occurrence of events can be undermined when, for example, people are told that their memory is incorrect (i.e., social feedback). Our scoping review shows that being provided with external evidence (e.g., media reports) and social feedback were main reasons for why retractors reduced belief in the occurrence of their recovered memories (Li et al., 2023; Ost, 2017). This result is interesting as it differs from research using student participants, where social feedback was mentioned as a reason that belief in occurrence of events is undermined (e.g., Scoboria et al., 2015). An explanation for this could be that retractors might have encountered, for example, newspaper articles regarding similar stories, which questioned the veracity of their accounts. Such examples are less likely to occur, of course, for more mundane events.

We also found that retractors differed on their belief and recollection of the event after their recantation (Li et al., 2023; Ost, 2017). Specifically, Ost (2017) showed that retractors accounts could be categorized along different memory types such as false beliefs or nonbelieved memories. For example, Ost found that some accounts even did not show any evidence of recollection and only contained signs of belief. Li et al. (2024), however, specifically asked retractors to rate their belief and recollection before and after their retraction. Interestingly, on average, belief scores were statistically lower after (versus before) retraction while this was not the case for recollection scores. These findings call for further research on belief-recollection dynamics in accounts of retractors (see also below).

### Limitations

A limitation of survey research using retractors is that they are based on self-reports. Because of this reliance on self-reports, it is of course unclear what the ground truth is in the accounts of retractors. This limitation has also been raised in critical commentaries on the topic of retractors (Reviere, 1997; Vos, 2024). Although it is true that in many retractor cases, no ground truth is available, the current findings map well with research on (1) how suggestions can distort memory (e.g., Loftus, 2005), (2) suggestive therapeutic practices (e.g., Schemmel et al., 2024), (3) therapeutic beliefs on the existence of repressed memory (e.g., Zappalà et al., 2024), and (4) how belief and recollection are impacted by various factors such as social feedback (e.g., Otgaar et al., 2014). Furthermore, from a legal point of view, the exoneration of the father of John Parker (see above) indicates that John’s memories were regarded as false indicating that there is evidence showing that retracted memories were originally false.

In our scoping review, we also encountered other critical articles on the subject of retractors. That is, the work described in this review is oftentimes based on a small pool of retractors which might limit the generalizability of the findings (Qin et al., 1997). The critique might not be easily addressed as it is unknown how large the total population of retractors is. It might well be the case that this group is not extremely large which would suggest that the current findings might be a reasonable representation of the entire population of retractors. Nonetheless, in a recent article^4^ (Kenny and Felstead, 2024), qualitative interviews were conducted with “only” three retractors in order to obtain more in-depth information than when a quantitative approach would be used. In this article, retractors indicated that they used different coping strategies to deal with their retraction such as education to understand what happened, forgiving the therapist, but also confronting the therapist.

An alternative way to address the issue of generalizability is not to test retractors of abuse per se, but to examine the prevalence of retracted memories of everyday events in the general population. Li et al. (2024) examined this specific issue in participants from China and other countries and found that around 50% (*n* = 698) and 30% (*n* = 166), respectively, reported to have had retracted memories in the past. Similar to research with retractors, social feedback was an important reason for retracting memories and belief scores were also much lower after (than before) withdrawal. These findings suggest that the way retraction of traumatic (recovered) memories occurs might be related to how memories are retracted in general.

Other critical articles focused on the observation that based on retractors’ accounts, it is difficult to know why retractors recovered memories in the first place (e.g., because of a narrative construction or a false belief existing before therapy; Gudjonsson, 1997; Singer, 1997). We agree with this issue, and it is, to a certain extent, connected to the first limitation. Specifically, research on retractors frequently relies on self-reports. Thus, when retractors describe what they experienced before, during, after therapy, they are basically attempting to retrieve a memory and such retrievals might not necessarily provide a fully accurate description of what happened. Although this issue is difficult to overcome, there are some cases in which notes of therapists were available which could then be compared with the accounts of the patients; sometimes providing additional evidence for the existence of suggestive therapeutic interventions (e.g., Otgaar et al., 2022b; Stridbeck, 2020).

### Concluding remarks

The topic of retractors is part of an ongoing debate on the existence of repressed memories and the authenticity of recovered memories (e.g., Lynn et al., 2023; Otgaar et al., 2019). To resolve this debate, research is needed on several fronts such as research into (1) therapeutic practices, (2) therapists’ beliefs about memory, and (3) effects of suggestion on memory. The research on these fronts has been ongoing and continues to show that suggestive therapeutic practices persist, as does the belief in repressed memories (e.g., Zappalà et al., 2024). What is, however, missing in this story is the input from research on retractors (see also Lynn et al., 2023). The aim of the current scoping review was to provide a timely overview of this research.

Our main conclusion is that based on retractors’ experiences and accounts, it appears that therapy played a vital role in recovering potentially false memories of abuse. These therapeutic practices were often based on a strong held belief in the existence of repressed memory. Furthermore, when they retracted their memories, either because they were told that their experience was fabricated or because they encountered external evidence, their accounts varied on belief and recollection. Our review calls for continued research on the topic of retractors such as the examination of whether they are especially susceptible to suggestion and the investigation of more in-depth interviews of the experiences of these retractors (see Kenny and Felstead, 2024). Such in-depth interviews might, for example, reveal whether the encountered suggestions during therapy might have affected their identity and self which could have affected their willingness to recover and later retract memories.

To conclude, combined with other research (e.g., Patihis and Pendergrast, 2019), our review adds to the notion that therapists believing in the concept of repressed memory might be likely to use suggestive interventions to unearth these memories leading to the formation of false memories. This issue encourages further research into how scientific knowledge of memory might make people less likely to use such suggestions in practice (e.g., Battista and De Beuf, 2024).

## Data Availability

All data can be accessed via https://osf.io/vuas7/.

## Footnotes

^1^See https://www.law.umich.edu/special/exoneration/Pages/casedetail.aspx?caseid=6587.

^2^We used the term “dissociative amnesia” as scholars have argued that it is similar to repressed memory (e.g., Battista et al., 2023; Otgaar et al., 2019).

^3^This website was used to calculate Cohen’s Kappa: https://www.graphpad.com/quickcalcs/kappa2/.

^4^This article was published after our search and hence, was not included in our review.
